# Health Care Providers’ Recommendations for Physical Activity and Adherence to Physical Activity Guidelines Among Adults With Arthritis

**DOI:** 10.5888/pcd10.130077

**Published:** 2013-11-07

**Authors:** Shamly Austin, Haiyan Qu, Richard M. Shewchuk

**Affiliations:** **Author Affiliations:** Shamly Austin, Department of Critical Care Medicine, University of Pittsburgh, Pennsylvania; Haiyan Qu, Department of Health Services Administration, University of Alabama at Birmingham, Alabama.

## Abstract

**Introduction:**

Physical activity is beneficial for reducing pain and improving health-related quality of life among people with arthritis. However, physical inactivity is prevalent among people with arthritis. Health care providers’ recommendations act as a catalyst for changes in health behavior. However, information about the effectiveness of such recommendations is limited in the arthritis literature. We examined the association between providers’ recommendations for physical activity and adherence to physical activity guidelines for adults with arthritis and whether adults’ age influenced this association.

**Methods:**

We used combined data of adult respondents aged 45 years or older with provider-diagnosed arthritis (N = 10,892) from the 2011 Behavioral Risk Factor Surveillance System to conduct a retrospective, cross-sectional study. We used a multivariable logistic regression model to examine the association between health care providers’ recommendations and adherence to physical activity guidelines among adults with arthritis.

**Results:**

Adults with arthritis who received health care providers’ recommendations for physical activity were more likely (odds ratio, 1.22; 95% confidence interval, 1.12–1.32) to adhere to physical activity guidelines than those who did not, after controlling for relevant covariates. Adults’ age did not influence the association between providers’ recommendations and adherence to physical activity (odds ratio, 1.00; 95% confidence interval, 0.99–1.00), after controlling for covariates.

**Conclusion:**

Health care providers’ recommendations are associated with adherence to physical activity guidelines among adults with arthritis. Providers should recommend physical activity to adults with arthritis.

## Introduction

Approximately 30 minutes of low- to moderate-intensity physical activity 5 days per week is recommended for people with arthritis (1,2). Adherence to physical activity guidelines has a positive association with health-related quality of life among people with arthritis (3). However, in 2004, fewer than 40% of US adults with physician-diagnosed arthritis adhered to the recommended levels of physical activity (4). One reason for the failure to follow these recommendations in this population may be lack of health care providers’ recommendations for physical activity (4–6).

Health care providers’ recommendations act as a catalyst for adherence to health-promoting behaviors (7,8). Studies on exercise, diabetes, weight loss, smoking cessation, and alcohol abuse treatment have reported that people who received providers’ recommendations for behavior changes were more likely to do so (9–12). Arthritis literature has limited information on the association between providers’ recommendations for physical activity and people’s adherence to physical activity guidelines. 

Not all people receive behavior change recommendations from their providers (13). A previous study found that older adults (aged 65 years or older) with arthritis were less likely to receive physical activity recommendations from their providers compared with their middle-aged counterparts (aged 45–64 years) (13). Providers’ lack of agreement with, familiarity with, awareness of, and low outcome expectancy of compliance with the guidelines are reported reasons for not recommending health promoting behavior changes (14). 

The first objective of our study was to examine the association between health care providers’ recommendations for physical activity and adherence to physical activity guidelines. Our second objective was to examine whether adults’ age influenced the association between providers’ recommendations for physical activity and adherence to physical activity guidelines in the arthritis population.

## Methods

### Data and sample

We used data from the 2011 Behavioral Risk Factor Surveillance System (BRFSS) survey, a random–digit-dialed landline and cellular telephone household survey of the noninstitutionalized civilian US adult population aged 18 or older, administered by the Centers for Disease Control and Prevention (CDC).

A 3-level framework (15) was used to ensure that all relevant factors associated with adherence to physical activity from the BRFSS data set were considered in the analysis. The framework includes individual-, interpersonal-, and environmental-level factors. The individual-level factors in our analysis were age, race, sex, education, income, employment, overall health status, obesity, activity limitations, and comorbidities. The interpersonal level factors were marital status, and health care providers’ recommendations; the environmental factors were issues related to access to care, such as access to health care coverage, having a personal physician, and region of residence.

The 2011 BRFSS arthritis management module was administered in 5 US states (Michigan, Minnesota, South Carolina, Tennessee, and Wisconsin) (16). Our sample included people aged 45 or older with health care provider–diagnosed arthritis. The study sample size was 10,908 respondents who had complete information on the key variables in the analysis model. When weighted to 2010 US population estimates, the final sample size included in the analysis was 10,892.

### Variables and measurements

The dependent variable was adherence to physical activity guidelines. The physical activity module of the BRFSS questionnaire records respondents’ self-reported physical activity in a usual week. On basis of the physical activity module, CDC derived a calculated variable known as “adherence to recommended levels of physical activity.” The measures for the variable were “adhered to the physical activity guidelines” and “did not adhere to the physical activity guidelines.” This classification was based on the American College of Rheumatology’s (ACR’s) recommendations for physical activity (1). People who adhered to physical activity guidelines reported at least 30 minutes of physical activity on 5 or more days per week. People who did not adhere to the physical activity guidelines included those who did not meet the recommended levels and those who were physically inactive.

The independent variable was health care providers’ recommendations for physical activity. The measure of the response was dichotomous (yes/no). Health care providers’ recommendations for physical activity for people with arthritis was measured as whether a physician or other health care professional recommended physical activity or exercise to help people’s arthritis or joint symptoms.

The control variables were measured as age (years, continuous variable); sex (male/female); race (white/nonwhite); education (≤high school diploma/>high school diploma); employed (yes/no); annual household income (<$50,000/≥$50,000); health status (good/poor); obesity (yes [body mass index ≥30 kg/m^2^]/no [body mass index <30 kg/m^2^]); activity limitations (yes/no); comorbidities (continuous variable); marital status (married/other); health care coverage (yes/no); personal physician (yes/no); and region of residence (Midwest and South). We computed comorbidities by summing the responses on diabetes, hypertension, high cholesterol, myocardial infarction, angina, stroke, asthma, and depression. All affirmative responses were coded as “1,” and negative responses were coded as “0,” such that comorbidities for an individual ranged from 0 to 8 when summed. Items from the BRFSS questionnaire are valid and reliable (17,18).

### Analysis

The institutional review board of the University of Alabama at Birmingham (protocol no. N090121006) approved this study. For data management and analyses, we used SPSS (SPSS, Inc, Chicago, Illinois) version 19.0. Univariate (frequency) and bivariate statistical tests were conducted to explore the data and test multicollinearity among the independent variables. Multivariable logistic regression model was used to analyze the association between health care providers’ recommendations for physical activity and adherence to physical activity guidelines controlling for the covariates in the model (Table 1). To examine the influence of age on the association between providers’ recommendations and adherence to physical activity guidelines, we examined the interaction term (age × providers’ recommendation for physical activity) in a multivariable logistic regression model. The Hosmer-Lemeshow test was used to assess the goodness of fit of the model. The results were presented as odds ratios (ORs), and significance was set at *P* < .05.

## Results

Overall, 49% respondents adhered to physical activity guidelines (Table 1). Only 60% received providers’ recommendations for physical activity. The average age of respondents was 64.6 years (standard deviation = 10.7).

**Table 1:** Factors Influencing Adherence to Physical Activity Recommendations, Adults With Arthritis (N = 10,892^a^), 2011 Behavioral Risk Factor Surveillance System

Variable		Overall, % (95% CI)	Adherence to Physical Activity Guidelines, % (95% CI)	
			Did Not Adhere (n = 5,520)	Adhered (n = 5,372)
**Interpersonal factors**				
**Received health care providers’ recommendations**				
Yes		59.4 (58.4–60.3)	58.8 (57.5–60.2)	59.9 (58.6–61.2)
No		40.6 (39.7–41.6)	41.2 (39.8–42.5)	40.1 (38.8–41.4)
**Marital status**				
Others		51.9 (51.0–52.9)	55.3 (54.0–56.6)	48.4 (47.1–49.8)
Married		48.1 (47.1–49.0)	44.7 (43.4–46.0)	51.6 (50.2–52.9)
**Individual factors**				
Age, y (SD)		64.6 (10.7)	64.1 (10.5)	65.1(10.5)
**Sex**				
Female		73.0 (72.2–73.8)	75.5 (74.5–76.6)	70.4 (69.2–71.5)
Male		27.0 (26.2–27.8)	24.5 (23.4–25.5)	29.6 (28.5–30.8)
**Race**				
Nonwhite		18.7 (17.9–19.4)	21.8 (20.6–22.9)	15.5 (14.5–16.5)
White		81.3 (80.6–82.1)	78.2 (77.1–79.4)	84.5 (83.5–85.5)
**Education**				
≤High school diploma		44.3 (43.4–45.3)	52.4 (51.1–53.8)	36.0 (34.7–37.3)
>High school diploma		55.7 (54.7–56.6)	47.6 (46.2–48.9)	64.0 (62.7–65.3)
**Employed**				
No		68.7 (67.8–69.6)	68.8 (67.6–70.1)	68.7 (67.4–69.9)
Yes		31.3 (30.4–32.2)	31.2 (29.9–32.4)	31.3 (30.1–32.6)
**Annual household income, $**				
<50,000		71.4 (70.6–72.3)	77.8 (76.7–78.9)	64.8 (63.5–66.1)
≥50,000		28.6 (27.7–29.4)	22.2 (21.1–23.3)	35.2 (33.9–36.5)
**Health status**				
Poor		32.2 (31.3–33.1)	42.3 (41.0–43.7)	21.8 (20.7–22.9)
Good		67.8 (66.9–68.7)	57.7 (56.3–59.0)	78.2 (77.1–79.3)
**Obese**				
Yes		39.4 (38.5–40.3)	46.3 (44.9–47.6)	32.3 (31.1–33.6)
No		60.6 (59.7–61.5)	53.7 (52.4–55.1)	67.7 (66.4–68.9)
**Activity limitation**				
No		48.1 (47.1–49.0)	40.8 (39.5–42.1)	55.5 (54.2–56.9)
Yes		51.9 (51.0–52.9)	59.2 (57.9–60.5)	44.5 (43.1–45.8)
**Comorbidities**				
0		11.6 (11.0–12.3)	8.8 (8.0–9.6)	14.6 (13.6–15.6)
1		24.3 (23.5–25.1)	21.6 (20.5–22.7)	27.0 (25.9–28.3)
2		27.5 (26.6–28.3)	27.5 (26.3–28.7)	27.4 (26.2–28.6)
3		19.8 (19.1–20.6)	21.3 (20.2–22.4)	18.3 (17.3–19.3)
4		9.6 (9.0–10.2)	11.7 (10.7–12.7)	7.5 (6.8–8.3)
5		4.8 (4.4–5.3)	6.0 (5.4–6.7)	3.6 (3.2–4.2)
6		1.8 (1.6–2.1)	2.4 (2.1–2.9)	1.1 (0.9–1.4)
7		0.5 (0.4–0.6)	0.6 (0.4–0.9)	0.4 (0.2–0.6)
8		0.1 (0.1–0.2)	0.1 (0.1–0.1)	0.1 (0.0–0.2)
**Environmental factors**				
**Health care coverage**				
No	7.8 (7.3–8.4)		9.1 (8.4–10.0)	6.5 (5.8–7.2)
Yes	92.2 (91.6–92.7)		90.9 (90.0–91.6)	93.5 (92.8–94.2)
**Personal physician**				
No	12.8 (12.2–13.5)		13.0 (12.1–13.9)	12.7 (11.9–13.7)
Yes	87.2 (86.5–87.8)		87.0 (86.1–87.9)	87.3 (86.3–88.1)
**Region**				
Midwest	61.2 (60.3–62.1)		56.2 (54.9–57.6)	66.3 (65.0–67.6)
South	38.8 (37.9–39.7)		43.8 (42.4–45.1)	33.7 (32.4–35.0)

Abbreviation: CI, confidence interval.

a Sample weighted to 2010 US population estimates.

Respondents who received providers’ recommendations for physical activity were more likely (OR, 1.22; 95% confidence interval [CI], 1.12–1.32) to adhere to physical activity guidelines than respondents who did not, controlling for the covariates in the model (Table 2). Among the covariates, all variables except marital status, respondent’s age, race, health care coverage, and having a personal physician were significant predictors of adherence to physical activity guidelines. Respondents who were unemployed (OR, 1.49; 95% CI, 1.35–1.65) and those residing in the Midwestern US region (OR, 1.34; 95% CI, 1.23–1.45) were more likely than employed respondents and respondents living in the South to adhere to physical activity guidelines; people with overall poor health (OR, 0.53; 95% CI, 0.48–0.59), who were obese (OR, 0.66; 95% CI, 0.61–0.72), and who had activity limitations (OR, 0.70; 95% CI, 0.64–0.76) and comorbidities (OR, 0.94; 95% CI, 0.91–0.97) were less likely than their counterparts to adhere to physical activity guidelines. Overall, employment and health status were the strongest predictors for adherence to physical activity guidelines. The Hosmer-Lemeshow goodness of fit test (*P* = .24) indicated that the model was a good fit.

**Table 2:** Association Between Health Care Providers’ Recommendations for Physical Activity and Adherence to Physical Activity Guidelines, Adults With Arthritis (n = 10,892^a^), 2011 Behavioral Risk Factor Surveillance System

Variable	OR (95% CI)
**Interpersonal factors**	
**Received health care providers’ recommendations**	
No	1 [Reference]
Yes	1.22 (1.12–1.32)^b^
**Marital status**	
Married	1 [Reference]
Other	1.00 (0.91–1.09)
**Individual factors**	
**Age, y**	1.00 (0.99–1.00)
**Sex**	
Male	1 [Reference]
Female	0.77 (0.70–0.85)^b^
**Race**	
White	1 [Reference]
Nonwhite	0.92 (0.82–1.02)
**Education**	
>High school diploma	1 [Reference]
≤High school diploma	0.61 (0.56–0.67)^b^
**Employed**	
Yes	1 [Reference]
No	1.49 (1.35–1.65)^b^
**Annual household income, $**	
≥50,000	1 [Reference]
<50,000	0.72 (0.64–0.79)^b^
**Health status**	
Good	1 [Reference]
Poor	0.53 (0.48–0.59)^b^
**Obese**	
No	1 [Reference]
Yes	0.66 (0.61–0.72)^b^
**Activity limitation**	
No	1 [Reference]
Yes	0.70 (0.64–0.76)^b^
**Comorbidities**	
No	1 [Reference]
Yes	0.94 (0.91–0.97)^b^
**Environmental factors**	
**Health care coverage**	
Yes	1 [Reference]
No	0.94 (0.81–1.10)
**Personal physician**	
Yes	1 [Reference]
No	1.04 (0.92–1.18)
**Region**	
South	1 [Reference]
Midwest	1.34 (1.23–1.45)^b^

Abbreviations: OR, odds ratio; CI, confidence interval.

a Sample weighted to 2010 US population estimates.

b *P <* .05.

Adults’ age did not influence the association between providers’ recommendations for physical activity and adherence to physical activity (OR, 1.00; 95% CI, 0.99–1.00) after controlling for covariates (Table 3). The Hosmer-Lemeshow goodness of fit test (*P* = .05) indicated that the model was a poor fit.

**Table 3:** Influence of Age on the Association Between Health Care Providers’ Recommendations for Physical Activity and Adherence to Physical Activity Guidelines, Adults With Arthritis (n = 10,892^a^), 2011 Behavioral Risk Factor Surveillance System

Variable	OR (95% CI)
**Interpersonal factors**	
**Received health care providers’ recommendations**	
No	1 [Reference]
Yes	1.22 (1.12–1.32)
**Marital status**	
Married	1 [Reference]
Other	1.00 (0.91–1.09)
**Demographic factors**	
**Age**	1.00 (0.99–1.00)
**Sex**	
Male	1 [Reference]
Female	0.77 (0.71–0.85)^b^
**Race**	
White	1 [Reference]
Nonwhite	0.92 (0.82–1.02)
**Education**	
>High school diploma	1 [Reference]
≤High school diploma	0.61 (0.56–0.67)^b^
**Employed**	
Yes	1 [Reference]
No	1.49 (1.35–1.65)^b^
**Annual household income, $**	
≥50,000	1 [Reference]
<50,000	0.72 (0.64–0.79)^b^
**Health status**	
Good	1 [Reference]
Poor	0.53 (0.48–0.59)^b^
**Obese**	
No	1 [Reference]
Yes	0.66 (0.61–0.72)^b^
**Activity limitation**	
No	1 [Reference]
Yes	0.70 (0.64–0.76)^b^
**Comorbidities**	
No	1 [Reference]
Yes	0.94 (0.91–0.97)^b^
**Environmental factors**	
**Health care coverage**	
Yes	1 [Reference]
No	0.94 (0.81–1.10)
**Personal physician**	
Yes	1 [Reference]
No	1.04 (0.92–1.18)
**Region**	
South	1 [Reference]
Midwest	1.34 (1.23–1.45)^b^

Abbreviations: OR, odds ratio; CI, confidence interval.

a Sample weighted to 2010 US population estimates.

b *P <* .05.

## Discussion

We found an association between providers’ recommendations for physical activity and adherence to physical activity guidelines among adults aged 45 or older who had arthritis. We also found that adults’ age did not influence this association. Moreover, our results indicate that people in poor health — those who were obese, who had an overall poor health status, and who had activity limitations and comorbidities — were less likely than people in good health to adhere to physical activity guidelines.

Respondents who received providers’ recommendations were more likely to adhere to physical activity guidelines than those who did not, after controlling for covariates. The rationale for why people follow providers’ recommendations for physical activity can be explained by Parsons’ traditional sick role perspective (19,20), which states that people respond to pain, discomfort, and overall sense of well-being. They consult health care providers when symptoms interfere with their ability to function in their daily activities (19) and seek providers’ care and cooperate with them in the process of recovery (20). The difference in knowledge between the health care providers and patients justifies both the providers’ assumption of authority and the patients’ trust, confidence, and norm of obedience (21). Hence, with the debilitating pain that interferes with their daily functions, people with arthritis are more likely to adhere to physical activity guidelines when they receive providers’ recommendations.

Although health care providers’ recommendations are important for adherence to physical activity, providers may not recommend physical activity to all people. Cabana and colleagues attribute this provider behavior to knowledge-related barriers such as lack of awareness or familiarity with the guideline, attitude-related barriers such as lack of agreement, and outcome expectancy or external/environmental barriers such as lack of time, resources, reimbursements, organizational constraints, or perceived increase in malpractice liability (14). Our results were similar to those of a previous study of people from 4 community-based family medicine clinics in southeastern Missouri on physicians’ recommendations and patients’ behavior changes in diet, exercise, and smoking cessation (7). In addition, both panel and cross-sectional studies in weight loss (11), smoking cessation (9), alcoholism treatment (10), and physical activity adoption (22) support our findings. However, to the best of our knowledge, this is the first time that the association between health care providers’ recommendations for physical activity and adherence to physical activity guidelines has been comprehensively analyzed in arthritis literature.

The ACR recommends 30 minutes of low- to moderate-level physical activity 5 days per week for people with all forms of arthritis (1,2,23). The low adherence to physical activity among people with arthritis can be addressed with providers’ recommendations in clinical settings. However, only 60% of respondents reported receiving providers’ recommendations for physical activity. Even among those who did not adhere to physical activity guidelines, 59% received providers’ recommendations’ for physical activity. Also, in our model, having a personal physician was not a significant predictor of adherence to physical activity guidelines. Hence, providers’ recommendations alone are not sufficient for people’s adherence to physical activity guidelines. Clinic-based intensive interventions such as the Five A’s — Ask, Advise, Assess, Assist, and Arrange, used in smoking cessation programs (24,25) — may be adapted to improve adherence to physical activity. Providers may ask patients about their engagement in physical activity and advise them about the benefits of physical activity during their visits. Providers can assess patients’ readiness to engage in physical activity and develop strategies to facilitate patients’ physical activity engagement. Furthermore, providers may assist patients in planning and including physical activity in their daily schedule. Finally, in every subsequent visit, providers may follow up on patients’ adherence to physical activity.

Contrary to previous research that indicated that adherence to physical activity decreased with advancing age (26,27), in our sample, age was not a significant predictor of adherence to physical activity. A reason for the difference may be that respondents in the previous studies were from general US population, but in our sample they were people with arthritis and belonged to only Midwestern and Southern US regions. Moreover, the association between provider’s recommendations and adherence to physical activity guidelines did not vary by levels of age. Thus, our results indicate that provider’s recommendations may act as catalyst for adherence to physical activity for people of all ages. The strongest predictor of adherence to physical activity guidelines was employment. We found that unemployed respondents were more likely to adhere to physical activity guidelines. One reason for this finding may be that unemployed people are more likely to lack health care coverage (28) and may have more out-of-pocket expenses for health care. Hence, financial concerns may drive these people to adhere to physical activity guidelines. Further research is required in this direction. Contrary to the findings from evidence-based research, the conventional belief is that engaging in physical activity might harm people’s joints and thus deteriorate their arthritis condition (23). Our results indicated that people who reported health conditions such as overall poor health status, obesity, and activity limitations and comorbidities were less likely to adhere to physical activity guidelines. Although benefits of physical activity have been documented for all forms of arthritis (1,2), health care providers need to play a key role in promoting physical activity among adults with arthritis, especially among people who have additional health conditions.

The study has several limitations. First, we could not infer causality because of the cross-sectional design of the study. Second, the arthritis management module of the 2011 BRFSS survey was administered in only 5 US states from Midwestern and Southern regions; therefore, our results may not be generalizable to Northeastern and Western US regions. Third, self-reported data are subject to desirability bias and recall bias and may result in underreporting or overreporting. Fourth, severity of pain varies widely among people with arthritis, depending on type of arthritis and amount of joint involvement, and we did not include pain as a covariate in our model. However, as a proxy for pain we included activity limitations; severity of pain is the strongest predictor of activity limitations (29). Fifth, BRFSS measured whether the provider recommended physical activity or not, but information was not available on whether the provider recommended 30 minutes of physical activity 5 times a week. Sixth, the nonwhite population accounted for a small percentage of our sample (African American: 9%, Hispanic: 3.1%, Asian/Pacific Islander /Native Indian/Alaskan/Multiracial: 3.4%), so our results may not be generalizable to racial/ethnic minorities. Seventh, other factors that may play a role in adherence to physical activity guidelines are past exercise behavior, time, motivation, and contextual variables such as availability of recreational facilities and neighborhood crime rates, for which we did not have any information.

Our results indicate that health care providers should be aware of the effect of their recommendations on patients’ adherence to physical activity guidelines and should promote physical activity engagement in clinical settings. Future research should focus on the influence of race/ethnicity on the association between providers’ recommendations and adherence to physical activity guidelines among people with arthritis and strategies to promote physical activity, especially in minority populations.
